# A Rapid Segmentation Method Based on Few-Shot Learning: A Case Study on Roadways

**DOI:** 10.3390/s25175290

**Published:** 2025-08-26

**Authors:** He Cai, Jiangchuan Chen, Yunfei Yin, Junpeng Yu, Zejiao Dong

**Affiliations:** School of Transportation Science and Engineering, Harbin Institute of Technology, Nangang District, Harbin 150006, China; 20b932024@stu.hit.edu.cn (H.C.); yunfeiyin@hit.edu.cn (Y.Y.); 22s132079@stu.hit.edu.cn (J.Y.); hitdzj@hit.edu.cn (Z.D.)

**Keywords:** road segmentation, unmanned aerial vehicles, back-projection, few-shot learning

## Abstract

Currently, deep learning-based segmentation methods are capable of achieving accurate segmentation. However, their deployment and training are costly and resource-intensive. To reduce deployment costs and facilitate the application of segmentation models for road imagery, this paper introduces a novel road segmentation algorithm based on few-shot learning. The algorithm consists of the back-projection module (BPM), responsible for generating target probabilities, and the segmentation module (SM), which performs image segmentation based on these probabilities. To achieve precise segmentation, the paper proposes a learning mechanism that simultaneously considers both positive and negative samples, effectively capturing the color features of the environment and objects. Additionally, through the workflow design, the algorithm can rapidly perform segmentation tasks across different scenarios without requiring transfer learning and with minimal sample prompts. Experimental results show that the algorithm achieves intersection over union segmentation accuracies of 94.9%, 92.7%, 94.9%, and 94.7% across different scenarios. Compared to state-of-the-art methods, it delivers precise segmentation with fewer local road image prompts, enabling efficient edge deployment.

## 1. Introduction

The development of computer vision technology has led to its increasingly widespread application in road systems. In computer vision, semantic segmentation is a crucial technology that can provide both semantic information and localization information for objects within an image. With the advancement of drone technology, its application in road vision systems is becoming increasingly widespread. The unmanned aerial vehicles (UAVs) can capture images of the road surface from multiple angles and heights, which can provide a comprehensive view of the condition of the road. In addition, UAVs can also reach all areas of the road without disrupting traffic, which have great potential for road inspection [1,2] and tasks such as post-disaster road reconstruction [3]. However, there are many background areas in the images captured by the UAVs, and only road areas are interested. The segmentation of roads is needed to obtain road knowledge for detection and maintenance [4].

Road segmentation involves dividing a digital image into segments or sections, where each segment represents a part of a road. The road network information can be extracted from remote sensing images to serve smart navigation, autonomous driving, smart cities and intelligent transportation [5,6,7]. In road region segmentation, traditional solutions achieve segmentation by extracting road image features based on manually designed algorithms, such as mathematical morphology [8], genetic algorithms [9], fuzzy connectedness [10], etc. These methods exhibit accuracy and efficiency in solving specific categories such as low-resolution SAR images or roads with linear characteristics. However, they highly dependent on specific models and assumptions, which limit the use of adaptability and stability.

With the rapid growth of the data scale, traditional model-driven methods are gradually becoming unable to meet the needs of big data applications [11]. In order to improve the accuracy of algorithms in different scenarios, many scholars begin to improve algorithms through the application of deep learning (DL). In 2015, Long et al. [12] replaced the fully-connected layer of CNN with the convolutional layer and proposed a fully convolutional neural network (FCNN) model. The proposal of FCNN has significantly advanced the development of DL for semantic segmentation. After that, Henry et al. [13] used FCNN to segment roads in TerraSAR images. Although it can successfully accomplish the segmentation task in the majority of scenarios at that time, it has exhibited sensitivity to forest boundaries. Since then, Deeplab [14], U-Net [15], and other proposed models [16,17] have also been successively applied to road segmentation. Overall, such end-to-end DL models can achieve automatic feature extraction and classification, thereby avoiding complex algorithm design and providing relatively high accuracy. However, the methods ignore spatial information and suffer from sample imbalance. Furthermore, the performance of DL is expected to decline when there is a shift in study domains, as indicated by Neupane et al. [18]. The existence of these drawbacks also limits the application of DL in other scenarios.

**Figure 1:**
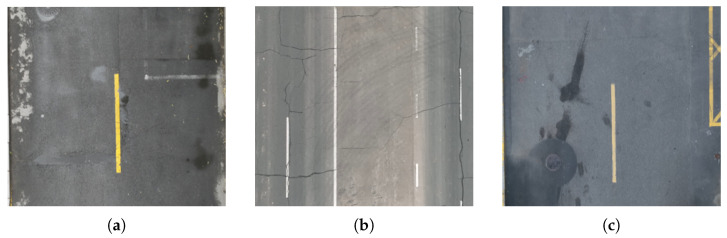
Road scenes in different environments, where differences in the color characteristics of pavements make it difficult to achieve accurate segmentation with a common model. (**a**) Pavement with puddles after rain. (**b**) Gray pavement with potholes due to long service life. (**c**) Renovated asphalt pavement.

Although deep learning-based methods have achieved notable accuracy in road segmentation tasks, their generalization ability across different scenes remains limited. In UAVs imagery, roads exhibit inhomogeneous colors and varying widths, often appearing unpaved, as shown in Figure 1. Consequently, segmentation methods tailored for specific types of roads may not be suitable for others [19]. In addition, constructing datasets for transfer learning requires a substantial amount of effort as well as computational resources. In summary, segmentation still remains difficult for different types of roads under variable lighting conditions.

Training and tuning the network for different application scenarios is time consuming and costly. Therefore, a more convenient method is proposed for the efficient segmentation of road images captured by UAVs in this paper. The segmentation of roads in a video can be achieved by learning from several image frames and be quickly adapted to different road scenes. The main contributions of this paper are as follows: An improved back-projection method that uses multiple positive and negative samples is proposed. The features of multiple samples can be integrated and eliminated by the different operators proposed in this paper. An easy-to-deploy road segmentation algorithm is designed to quickly adapt and realize road video segmentation for different environments. The algorithm combines the color features and morphological connectivity features of roads, which enables the accurate segmentation of roads at different scales of images.

The rest of this paper is organized as follows: Section 2 reviews related work relevant to the proposed algorithm; Section 3 details the design of the learning mechanism and parameter optimization process; Section 4 presents experiments applying the algorithm under various parameters and environments; finally, Section 5 concludes the paper and discusses potential application scenarios.

## 2. Related Works

### 2.1. Road Segmentation Method

The primary task of road segmentation is to utilize computer vision techniques to analyze the entire image and extract the road regions, thereby providing scene environment information for downstream tasks. Relevant technologies can be broadly categorized into traditional segmentation methods based on image features and end-to-end segmentation approaches driven by deep learning. Their application scenarios include remote sensing imagery [20], autonomous driving [21], and surveying and mapping [22].

Traditional road segmentation methods primarily rely on image processing techniques and probabilistic graphical models with the core idea of segmenting regions based on low-level visual features of pixels. These methods can be mainly categorized into threshold- and edge-based approaches [23], clustering- and graph theory-based methods [24], as well as implementations such as random decision forests and contour detection [25]. While these methods offer high computational efficiency, their generalization ability in complex scenes is limited, making them suitable primarily for relatively simple tasks today.

With the advancement of deep learning technology, convolutional neural networks, owing to their hierarchical feature extraction capabilities, have become the dominant architecture for road segmentation. Many leading semantic segmentation models have been adapted and applied to road image segmentation. For example, Zhang et al. [26] introduced a boundary-constrained multi-scale segmentation method based on U-Net tailored for remote sensing images, focusing on enhancing image analysis for land use classification. Xiao et al. [27] proposed a novel C-DeepLabV3+ algorithm that incorporates a Coordinate Attention module and a Cascade Feature Fusion module to improve road segmentation accuracy in UAV aerial images.

In recent years, with the introduction of the Transformer architecture, networks have been able to overcome the local receptive field limitations of CNNs through global modeling capabilities. Based on this, many researchers have incorporated attention mechanisms into segmentation models, significantly enhancing robustness in complex road scene segmentation. For example, Wu et al. [28] proposed TC-Net, a lightweight Transformer–Convolutional network for real-time road segmentation that employs Transformer-Conv and PatchMerging-Conv modules to reduce parameters while maintaining accuracy on the KITTI dataset. Tao et al. [29] introduced Seg-Road, a Transformer–CNN segmentation network for remote sensing road extraction that integrates a pixel connectivity structure to reduce fragmentation, achieving 67.2% IoU on DeepGlobe and 68.4% IoU on Massachusetts.

Deep learning-based road segmentation methods exhibit strong generalization capabilities, enabling extensive applications in scenarios such as aerial remote sensing and autonomous driving. However, for certain low-altitude scenarios where the overall image features vary complexly, fine-tuning networks to adapt to diverse conditions is time consuming and labor-intensive. Additionally, the substantial computational overhead of deep networks limits their deployment in real-time inference and edge computing environments. Therefore, there is a need to develop a road surface segmentation algorithm with low computational cost that facilitates deployment and transferability.

### 2.2. Few-Shot Semantic Segmentation

Traditional fully supervised semantic segmentation methods rely on large amounts of precisely annotated training data, with the annotation process being both time consuming and costly. Moreover, these models are typically limited to the categories present in the training set and struggle to generalize to new classes. Few-shot semantic segmentation has emerged as a novel research direction to address these challenges, enabling models to quickly adapt to and segment new categories using only a small number of annotated samples [30].

Current mainstream approaches can be categorized into two major technical routes: prototype-based learning and affinity-based learning. Prototype-based methods compress support features into class prototypes via masked average pooling and then perform segmentation by comparing these prototypes with query features, offering computational efficiency but suffering from spatial information loss and insufficient context awareness [31]. Affinity-based methods construct pixel-level feature matching directly, preserving fine-grained spatial relationships, but they are susceptible to background interference and may cause semantic ambiguity [32]. To overcome these limitations, recent research has begun exploring hybrid architectures that combine the strengths of both approaches. For example, Zhang et al. [33] proposed integrating Model-Agnostic Meta-Learning with SegNet and U-Net for the few-shot semantic segmentation of buildings and roads in remote sensing imagery.

**Figure 2:**
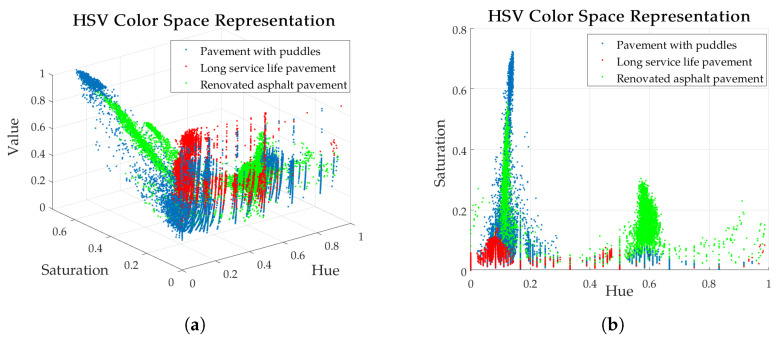
Distribution of pixels in three types of scenes; pavements are most distinguishable in the H and S color space. (**a**) Distribution of pixels in HSV color space. (**b**) Distribution of pixels in H and S space.

Although deep learning-based few-shot segmentation methods perform well in data-scarce scenarios, their inherent model complexity may result in excessive computational resource consumption, leading to unnecessary efficiency loss in simple image segmentation tasks [34]. HSV is a color model based on human visual perception, consisting of three dimensions: hue (H), saturation (S), and value (V). Roads in images often exhibit a regular color distribution, as shown in Figure 2; even across different scenes, their distribution in the HSV color space maintains a certain pattern. Based on this characteristic, road segmentation can be achieved at low cost by extracting the color distribution through back-projection.

Based on this characteristic, this paper innovatively proposes a color-learning mechanism that simultaneously considers both positive and negative samples, effectively extracting the color features of the road while eliminating environmental interference. Furthermore, addressing the specific features of road scenes, a mask growth algorithm is introduced to further optimize the segmentation results and enhance the algorithm’s performance. Finally, these improvements are integrated into a segmentation algorithm workflow. The algorithm boasts low floating-point computational complexity and ease of deployment, enabling rapid segmentation across different scenarios with minimal sample prompts and without the need for transfer learning.

## 3. Road Segmentation Based on Improved Back-Projection Algorithm

**Figure 3:**
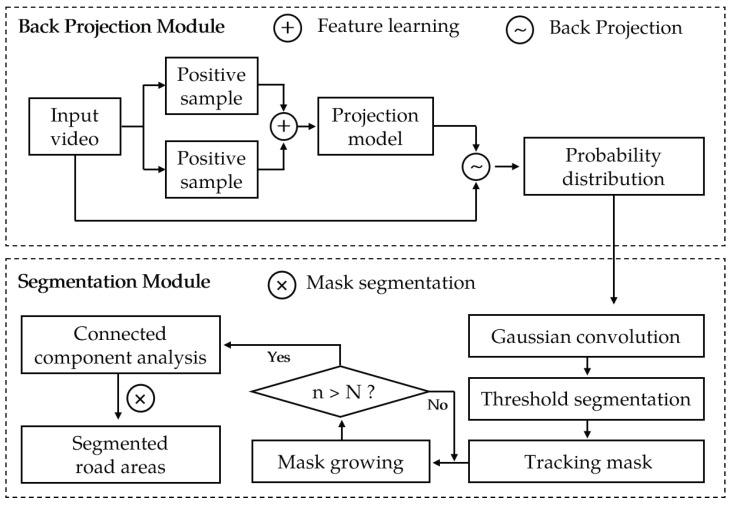
Algorithm flow of road projection segmentation.

The traditional back-projection algorithm has poor generalization and low accuracy, making it difficult to apply in road segmentation tasks. In this section, a learning mechanism is proposed to improve the generalization ability of the algorithm by learning multiple positive and negative images. Based on the improved learning mechanism, an algorithmic framework for road surface segmentation through few-shot learning has been designed and proposed, as illustrated in Figure 3.

The algorithm consists of two modules: a back-projection module (BPM), which is responsible for feature extraction and learning, and a segmentation module (SM), which realizes image segmentation based on feature distribution. In the BPM, the segmented video is first sampled, containing positive samples that include the road area and negative samples that include the road environment. After sampling, the histograms of the color distributions of the two types of samples are fused to obtain the projection model. Then, all frames within the video are back-projected to obtain the target distribution probability information. The role of the SM is to use the feature distribution obtained from the BPM for road segmentation. First, the features are Gaussian convolved and binarized using an adaptive threshold to obtain a segmentation mask. Then, the mask undergoes N-times of region growth. Voids are filled to obtain the main body of the road through CCA. Finally, the mask is multiplied with the road image to obtain the segmentation result. The details of each part will be described in the following subsections.

### 3.1. Histogram Learning Mechanism

In order to make the model close to the color characteristics of the segmented video, it is recommended that the samples be selected using a portion of the key frames in the video or a similar pavement image. Positive samples are selected from the image blocks of the road area, while negative samples are selected from the outside area. The colors of samples are converted from the RGB to HSV color space. Then, a 2D histogram is constructed with H and S values of the remainder. Given the total number of histogram bins in the H and S dimensions as M and N, respectively, the computation of the 2D histogram is achieved through the following equation:

(1) $$\begin{array}{cc} \; & H\left(h,s\right)=\sum_{i,j\in \Omega }\left(\delta^{2}\left(b_{h}\left(i,j\right)-h,b_{s}\left(i,j\right)-s\right)\right),\\ \; & \;0<h\leq M,\;0<s\leq N, \end{array}$$

where $b_{h}\left(i,j\right)=\left\lfloor \frac{p_{h}\left(i,j\right)}{\max\left(p_{h}\left(i,j\right)\right)}M\right\rfloor ,\;b_{s}\left(i,j\right)=\left\lfloor \frac{p_{s}\left(i,j\right)}{\max\left(p_{s}\left(i,j\right)\right)}N\right\rfloor .$

In the above equation, H(h,s) represents the value of the 2D histogram in the *h* interval of hue and *s* interval of saturation; p(i,j) denotes the H and S values of the pixel located at coordinates (i,j) respectively; the notation $\left\lfloor \cdot \right\rfloor$ indicates that the values within the brackets are rounded down to the nearest integer and Ω indicates the coordinate area of the sample.

After obtaining all the sample histograms, the next step involves learning about the color distribution among the samples. For positive samples $H_{t1}$ and $H_{t2}$, the features are merged using intersection and union operations. The equation for the intersection operation is

(2) $$\mathrm{Intersection}\left(H_{t2},H_{t1}\right)=\frac{\sum_{i,j\in \Omega }\min\left(H_{t1}\left(i,j\right),H_{t2}\left(i,j\right)\right)}{\min(\max\left(H_{t1}\right),\max\left(H_{t2}\right))}.$$

The intersection operation preserves similar characteristics among positive samples, enabling the compression and refinement of feature information within the samples. Consequently, this process reduces the impact of debris or shadows on road segmentation across diverse samples. The equation for the union operation is

(3) $$\mathrm{Union}\left(H_{t2},H_{t1}\right)=\frac{\sum_{i,j\in \Omega }\max\left(H_{t1}\left(i,j\right),H_{t2}\left(i,j\right)\right)}{\max(\max\left(H_{t1}\right),\max\left(H_{t2}\right))}.$$

The union operation can extend the features among samples, enhancing the generalization capability of the model. Additionally, this operation consolidates diverse road surface features, enabling the segmentation model to concurrently finish multiple segmentation tasks across varied scenarios.

For positive sample $H_{t}$ and negative sample $H_{f}$, the subtraction operation serves to adjust the features, eliminating those encompassing environmental color attributes within the samples. The equation for the subtraction operation is

(4) $$\mathrm{Subtraction}\left(H_{t},H_{f}\right)=\frac{\sum_{i,j\in \Omega }\max\left(\left(H_{t}\left(i,j\right)-H_{f}\left(i,j\right)\right),0\right)}{\max(\max\left(H_{t1}\right)-\max\left(H_{t2}\right),0)}.$$

The subtraction operation is able to remove the same color features from the road and the environment, making the segmentation result more accurate.

Through the above three types of operations, the general characteristics of the road can be learned from the samples of the same scene by intersection operations. Also, the model can learn from the samples from different scenes to realize segmentation in different types of roads by union operations. For the wrong results of the segmentation, the use of subtraction operations can be used for the removal of the interference color in the road, making the segmentation results more accurate.

**Figure 4:**
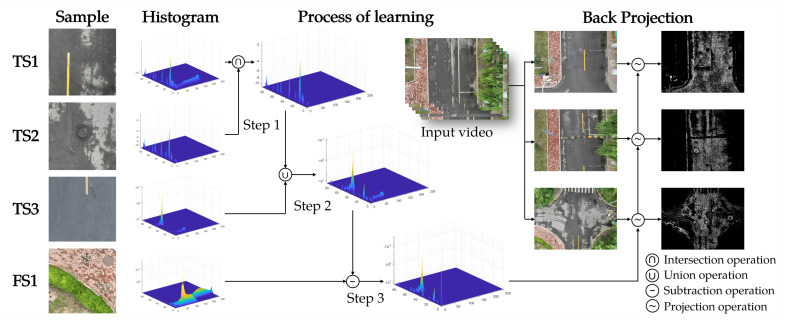
Process of the back-projection module.

In the BPM, its basic calculation process is shown in Figure 4. Firstly, some of the road images are selected as positive samples from the input video or similar road images. The features are merged using an intersection and union operation between the sample histograms. Then, a portion of the environment image is selected from the input video as negative samples. A subtraction operation is used on the positive samples that have been learned to finally obtain the projection model. After that, the project is modeled to the video to finally obtain the segmentation probability of the road.

### 3.2. Mask Generation and Growth

In BPM, the target probability distribution in discrete form is obtained. However, the regional probability distribution is needed to realize the segmentation of road; thus, the probability needs to be regionally weighted. In the application of the back-projection to the tracking task, the mean-shift algorithm is often used to extract the probability distribution of the region and find the target for tracking in the form of a sliding window [35]. Similarly, in order to give higher weights to the pixels close to the center, this paper uses the Gaussian convolution method to replace the sliding window for regional probability extraction. After completing the convolution operation, a road map is found according to the mean and variance of the Gaussian distribution. The road map is calculated as

(5) $$\mathrm{Map}\left(i,j\right)=\left\{\begin{array}{cc} 1, & P(i,j)\geq \mu -\beta \cdot \sigma \\ 0, & \mathrm{otherwise} \end{array}\right.,$$

where μ and σ represent the variance and mean of the distributional probabilities P(i,j); β is a parameter controling the threshold range. Note that the larger it is, the more pixels will be retained, while the accuracy decreases.

However, it is difficult for the map obtained by global mean and variance to reflect local color features. Certain shadows, dust, and other conditions can cause local color distribution zones to shift, resulting in voids of mask. These voids can be resolved through mask growth. The basic idea of the mask growth is to calculate the data distribution in the region, using a sliding window and expanding the mask in areas near the neighborhood of the average value, which can be written as

(6) $$G\left(\mathrm{Map},W\right)=\left\{\begin{array}{cc} 1, & \mathrm{if}|M\bar{a}p\left(x,y\right)\cdot P-\bar{\mu }|\leq \beta \cdot \bar{\sigma }\\ \; & \;\mathrm{for}\;(x,y)\;\mathrm{in}\;W\\ 1, & \mathrm{if}\;\mathrm{Map}(x,y)=1\\ 0, & \mathrm{otherwise} \end{array}\right.,$$

where $\bar{\mu }=\frac{\sum_{x,y\in W}\mathrm{Map}\left(x,y\right)\cdot P}{\mathrm{Sum}(\mathrm{Map}(x,y))},\bar{\sigma }=\sqrt{\frac{\sum_{x,y\in W}{\left(\mathrm{Map}\left(x,y\right)\cdot P-\bar{\mu }\right)}^{2}}{\mathrm{Sum}(\mathrm{Map}(x,y))-1}}.$

In the above equation, *W* is a sliding window of size a×a; (x,y) are the coordinates of the window; $\bar{\mu }$ is the probability mean of the intersection of *W* and $\bar{\sigma }$ represents the variance. In the growth of the map, first, the mean and variance of the probability of retained pixels within the window are calculated. Then, the pixels not in the map whose probability is within β times the variance of the mean are retained, and their corresponding maps are filled. Finally, the whole process is repeated N times to expand the map.

### 3.3. Connected Component Reservation and Filling

**Figure 5:**
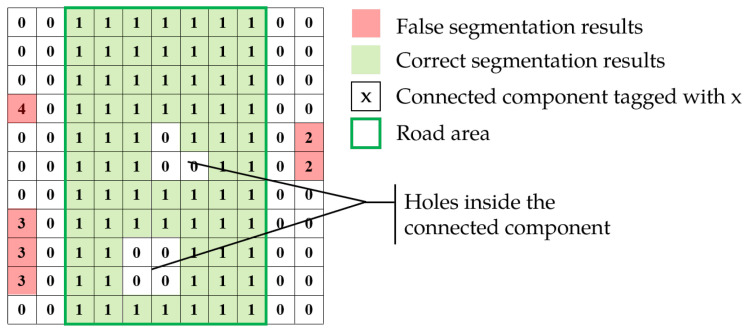
Connected component analysis label results.

Through the probabilistic projection, mask generation and growth, the basic segmentation masks of the road are obtained. However, due to the complex color environment, the masks tend to have the holes inside the road and some false segmentation results, as shown in Figure 5. In order to solve this problem, CCA is used to fill the holes and remove discrete regions. The forward scan mask is introduced to combine components of the road area of the image.

Through CCA, the connectivity regions can be obtained in the image. Meanwhile, the different connectivity regions are labeled with different labels. The road area mask can be obtained by counting each label and keeping the highest number one, which can be written as

(7) $$\mathrm{Mask}=\left\{\begin{array}{cc} 1 & \;\mathrm{if}\;\mathrm{label}=\mathrm{argmax}({\mathrm{label}}_{count})\\ 0 & \;\mathrm{otherwise} \end{array}\right.,$$

where ${\mathrm{label}}_{count}$ is the number of labels counted by number and argmax denotes the label corresponding to the maximum value from ${\mathrm{label}}_{count}$. The acquisition rule for the mask is to keep the pixels corresponding to the labels with the largest share in each image to obtain the segmentation of the road.

After obtaining the connected component, contour finding is performed on it, and the computational method proposed by Suzuki et al. [36] is chosen here. The algorithm first performs a raster scan of the binary image on the input to find the border following starting points. Then after that, it performs boundary tracking to find the complete boundary. The boundary is filled to obtain the road segmentation mask without the voids through the boundary search.

**Figure 6:**
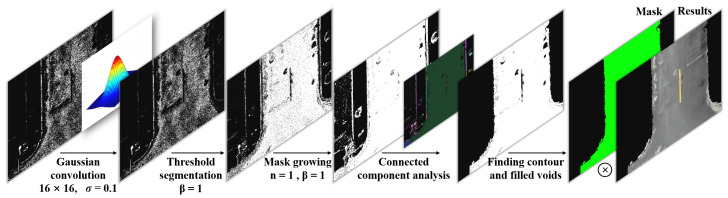
Process of the segmentation module.

Through the connected component reservation and filling, the image mask is finally obtained to realize the road image segmentation. In the SM, its basic algorithm processing flow is shown in Figure 6. Firstly, Gaussian convolution is performed on the probability distribution information obtained in BPM to extract the probability distribution features of the zones. Then, adaptive threshold and region growth are used to eliminate the influence of the brightness of the region on the segmentation accuracy. Finally, CCA is used to retain the maximum connectivity domain. Contours will be extracted and the voids within will be filled, which is followed by segmenting the image using the filled mask to achieve road segmentation.

### 3.4. Parameter Optimization Methods

In order to obtain the segmentation probability distribution, it is necessary to determine the HS histogram scale as well as the threshold coefficients for classification. Appropriate parameters can improve color differentiation in projection, improving the accuracy of segmentation. The value of parameters can be determined by calculating the segmentation effect indicator.

The scale of the histogram includes the number of M and N. To evaluate the performance of the algorithm, manual segmentation is used to acquire the binary mask and ground truth (GT) of the road. For the obtained segmentation probabilities, P(i,j), the performance of BPM is evaluated by the distinction (DT) obtained by calculating the probability sum in the correct region, $\Omega_{T}$, and the background region, $\Omega_{F}$. The DT can be calculated as

(8) $$\mathrm{DT}=\frac{\sum_{i,j\in \Omega_{T}}P\left(i,j\right)}{\sum_{i,j\in \Omega }P\left(i,j\right)}+\frac{\sum_{i,j\in \Omega_{T}}P\left(i,j\right)}{\sum_{i,j\in \Omega_{\mathrm{GT}}}\mathrm{GT}},$$

where $\Omega =\Omega_{T}+\Omega_{F}.$

It should be noted that in (8), the first part represents the recognition accuracy of the model, where the small probability or area of incorrect segmentation means a large value. The second term represents the recall of the probability, which is used to represent the model’s recognition of pavement areas.

In the SM, the threshold of segmentation is mainly determined by β, which is obtained based on the assumption that the overall probability conforms to a Gaussian distribution. The determination of β can be achieved by calculating intersection over union (IoU). The IoU value estimates the amount of overlap between the segmentation area and marker area (ground truth area), which can assess the correctness of a prediction as well as the rationality of β.

More precise results can be obtained by means of parameter tuning. The optimal parameters intervals are confirmed by calculating the indicator with different values, being suitable for most scenarios at the same time.

## 4. Experimental Results

**Figure 7:**
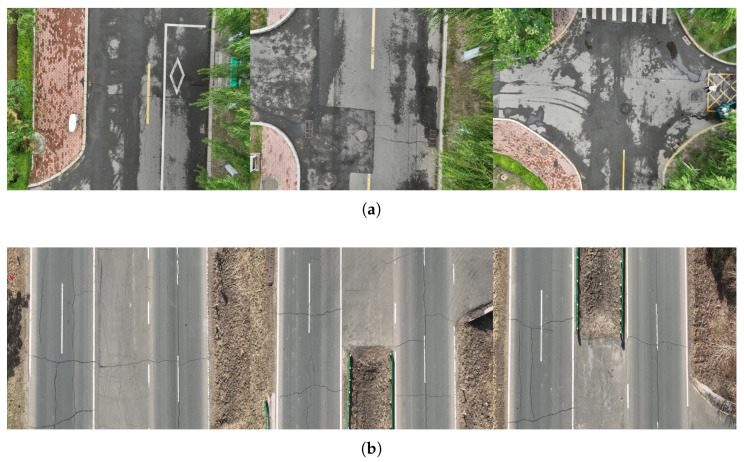
The tested videos collected in different scenarios. (**a**) Images taken over school roads with shadows of water. (**b**) Images taken over country roads with environmental disturbance.

The experimental data were collected using a DJI Mavic 3 drone. The collection sites included urban roads and suburban highways in Heilongjiang Province. Each video segment covered a distance of 1 km with a flight altitude of 20 m. Videos were sampled at equal time intervals, resulting in 20 images per scene category for validating the segmentation performance of the algorithm. For each scene, four pixel blocks were selected for environmental feature learning, and ground truth road surface regions were manually annotated to evaluate the algorithm’s segmentation accuracy. Both tested scenarios have the effect of environmental disturbances or shadows of water, as shown in Figure 7. The proposed algorithm has been tested in the acquired data. In each video, several parts of the road area from one or two frames are taken for sampling and model learning.

### 4.1. Experimental Parameter Optimization

**Figure 8:**
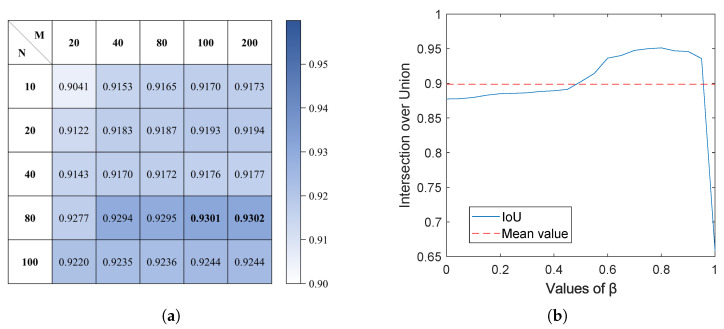
Segmentation effect under different parameters. (**a**) Calculation of DT. (**b**) Calculation of IoU.

To enhance segmentation effectiveness, refined parameters were meticulously selected for distinct scenarios based on the methodology outlined in Section 3.4. As can be seen in Figure 2, the road color space has a greater differentiation on hue, showing a concentrated distribution around 0.15 and 0.6 in the selected scene while having more coverage domains on saturation. Taking this into account, more bins such as 20, 40, 80, 100, and 200 were chosen for the values of M, while 10, 20, 40, 80, and 100 were chosen for N. DT is calculated under the combinations of these values, and the results are shown in Figure 8a. At different values of (M, N), DT achieves a better performance without exhibiting significant variations as M increases at (100, 80) in the selected scene. Considering that fewer partitions can achieve better generalization and less computation, the recommended values of M and N in this paper are M = 100 and N = 80.

In order to select the suitable value of β, it is sampled at intervals of 0.05 from 0 to 1 in this paper, calculating IoU at different values. From Figure 8b, it can be seen that when the value of β is between 0.6 and 0.9, the IoU is stable at about 0.95, which has good segmentation ability. When the value of β is too small, indicating a high segmentation threshold, it results in a reduced area for road recognition. On the contrary, a low segmentation threshold may result in inaccurate recognition. The recommended value of β in this paper is 0.65.

### 4.2. Algorithm Testing Under Complex Environment

**Figure 9:**
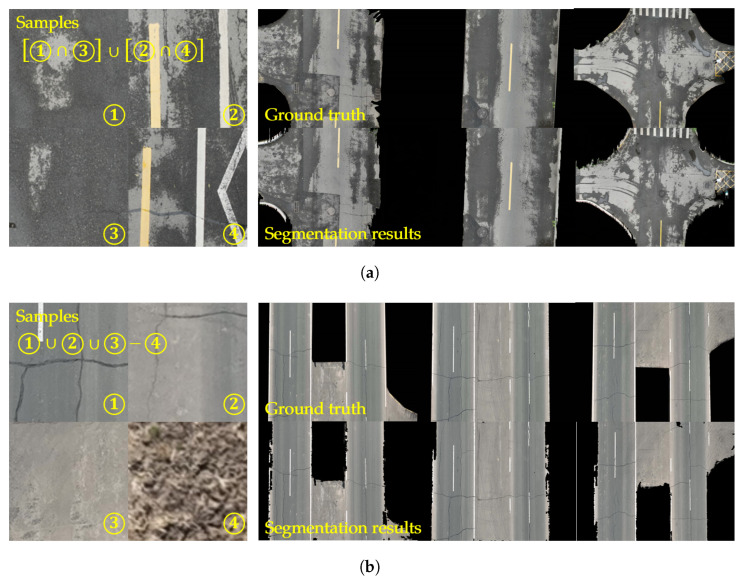
Segmentation effect of the algorithm in different scenarios. (**a**) Segmentation effect under wet road surface. (**b**) Calculation of IoU with different parameters.

In the road scene segmentation task, there are many interferences, most of them come from environment and the texture of road itself. Therefore the algorithm must have anti-interference ability in these scenes. In Figure 9a, the first scene detected is roads with puddles, accompanied by shadows cast due to the presence of water on the road. Four areas of the road are selected in two frames to extract road features.

These samples are crossed through the intersection operation, which is accompanied with the union operation to merge the features to obtain the projection model. The obtained model is used to segment the rest of the frames in the video by the algorithm. The average IoU is 94.89%, and the average precision is 92.73% in 10 frames of different scenes from videos. In Figure 9b, the detection scene is roads with similar color to the environment. Three areas of the road and one area of the environment in one frame are selected, which are followed by the union operation to merge the features of the road. Finally, the subtraction operation is used to remove the same features in the road as in the environment. The average IoU and precision in the segmentation of the remaining frames is 94.89% and 94.73%.

In the experiment, more than 10 images not containing the same scene are extracted from each video. The ground truth values in these images are obtained through manual segmentation while removing areas obscured by trees in the road. The test results are shown in Table 1. From the results, it can be seen that just by learning from the four samples of roads and environments, images can achieve good results in video segmentation, and such advantages can greatly reduce the deployment and training time in UAV road inspection and road area extraction, etc. Meanwhile, as shown in Figure 9a, some misidentified environmental regions appear along the road edges when negative samples are not selected. In Figure 9b, the introduction of the subtraction operation effectively mitigates the issue of background misidentification. Therefore, by covering as many features of the road color as possible, the accuracy of segmentation can be improved.

### 4.3. Comparative Experiments

Considering the application scenario of this algorithm under small sample learning, the traditional back-projection (BP) algorithm and automatic segmentation of the segment anything model (SAM) [37] are tested. Roads with puddles are chosen for the test scenario.

The tested BP algorithm is a wrapper function within the opencv library. Road segmentation is realized only by convolution and thresholding; meanwhile, the samples are selected from the road mask of one frame in the video, as shown in Figure 9a. The results show that the probabilistic discretization through single template projection greatly affects the accuracy of segmentation with 21.39% average IoU and 21.43% average precision, as shown in Table 2. Compared to our algorithm, the traditional BP method lacks a mechanism for learning the color distribution across multiple road regions, making it difficult to simultaneously extract color features from different parts of the road area and resulting in numerous voids in the detection outcomes. Additionally, the absence of convolutional steps restricts it to performing segmentation based solely on discrete data distribution rather than from an overall probabilistic perspective.

**Figure 10:**
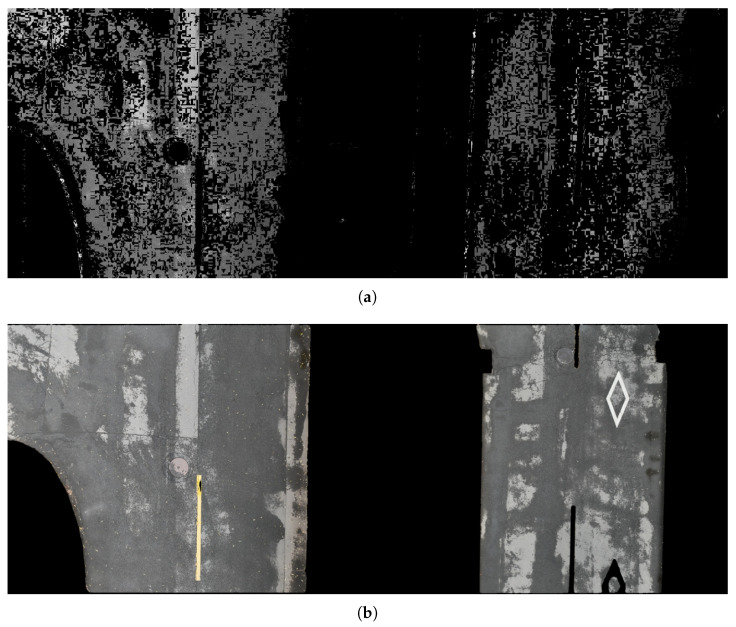
Segmentation effect of different models. (**a**) Segmentation effect of the traditional BP algorithm. (**b**) Segmentation effect of the SAM.

Since deep learning-based few-shot segmentation methods do not incorporate a negative sample learning mechanism, for the experiments involving SAM, we only used positive samples as prompts, which is consistent with our algorithm. SAM acquires scene semantic understanding through training on large-scale datasets, enabling few-shot segmentation with minimal prompts. The experimental results of SAM and SAM 2 on the same validation data are shown in Figure 10b. As illustrated, the scene understanding capability of SAM allows it to produce smoother edges. However, in road scenes with complex color textures, its performance is affected by road markings and water stains. Although this issue can be addressed through transfer learning, it requires substantial data and computational resources. While SAM 2 demonstrates certain advantages in overall segmentation metrics, achieving an IOU of 0.9692 and accuracy of 0.9813, its high computational demand limits its deployment on edge devices.

The algorithm was executed on an Intel(R) Core(TM) i5-10400F CPU using the MATLAB platform without involving GPU computation or complex environmental deployment. During operation, the algorithm only includes Gaussian convolution and morphological operations. At the same input resolution (1024 × 1024), the FLOPs of SAM differ by an entire order of magnitude compared to the algorithm proposed in this paper. Meanwhile, the algorithm contains no learnable parameters and does not rely on GPU computation. In dataset testing, the average inference time per image was 55.4 ms. These design advantages enable the algorithm to be easily deployed on most edge computing devices.

## 5. Conclusions

Considering the difficulty of training and deploying deep learning networks in tasks of road area extraction and segmentation in low-altitude UAV viewpoints, a small-sample road video segmentation method is proposed based on few-shot learning. To address the problems regarding the traditional back-projection algorithms’ poor generalization ability and inability to adequately represent the road color information, this paper proposes the feature learning mechanism to achieve the feature fusion and subtraction of environment color. The road is segmented by designing the algorithm. Experiments demonstrate that the algorithm is capable of realizing the task of segmenting road videos in different environments by just learning from several road area images. Also, the algorithm has a huge improvement over traditional back-projection.

The proposed algorithm can also be used for some segmentation tasks of the objects with distinctive color features, such as smoke, sky and leaves. Compared with the DL methods in different environments at high resolution, the proposed algorithm proves simpler to implement and remains unaffected by image texture. In summary, this paper expects to achieve efficient segmentation by extracting target features, and the obtained results can serve as a priori knowledge to direct UAVs toward areas of interest during road inspections.

## Figures and Tables

**Table 1:** Segmentation results for different road scenes.

Scenes	Minimum IoU	Minimum Precision	Average IoU	Average Precision
Roads with puddles	0.8437	0.8633	0.9273	0.9489
Roads with similar color	0.9277	0.93022	0.9473	0.9489

**Table 2:** Comparison of segmentation results between different models.

Scenes	Ave IoU	Ave Precision	FLOPS	Parameters (M)
Proposed algorithm	0.9273	0.9489	146 M	0.008
Traditional algorithm	0.2139	0.2143	-	-
SAM	0.9685	0.9809	746.4 G	93.7
SAM2	0.9692	0.9813	533.9 G	80.8

## Data Availability

The raw data supporting the conclusions of this article will be made available by the authors on request.
